# Genes Whose Gain or Loss-Of-Function Increases Skeletal Muscle Mass in Mice: A Systematic Literature Review

**DOI:** 10.3389/fphys.2018.00553

**Published:** 2018-05-22

**Authors:** Sander A. J. Verbrugge, Martin Schönfelder, Lore Becker, Fakhreddin Yaghoob Nezhad, Martin Hrabě de Angelis, Henning Wackerhage

**Affiliations:** ^1^Exercise Biology Group, Faculty of Sport and Health Sciences, Technical University of Munich, Munich, Germany; ^2^German Mouse Clinic, Institute of Experimental Genetics, Helmholtz Zentrum München, Neuherberg, Germany; ^3^Chair of Experimental Genetics, School of Life Science Weihenstephan, Technische Universität München, Freising, Germany; ^4^German Center for Diabetes Research (DZD), Neuherberg, Germany

**Keywords:** skeletal muscle, hypertrophy, gene manipulation, mutation, GWAS, sarcopenia, myostatin, resistance exercise

## Abstract

Skeletal muscle mass differs greatly in mice and humans and this is partially inherited. To identify muscle hypertrophy candidate genes we conducted a systematic review to identify genes whose experimental loss or gain-of-function results in significant skeletal muscle hypertrophy in mice. We found 47 genes that meet our search criteria and cause muscle hypertrophy after gene manipulation. They are from high to small effect size: *Ski, Fst, Acvr2b, Akt1, Mstn, Klf10, Rheb, Igf1, Pappa, Ppard, Ikbkb, Fstl3, Atgr1a, Ucn3, Mcu, Junb, Ncor1, Gprasp1, Grb10, Mmp9, Dgkz, Ppargc1a (specifically the Ppargc1a4 isoform), Smad4, Ltbp4, Bmpr1a, Crtc2, Xiap, Dgat1, Thra, Adrb2, Asb15, Cast, Eif2b5, Bdkrb2, Tpt1, Nr3c1, Nr4a1, Gnas, Pld1, Crym, Camkk1, Yap1, Inhba, Tp53inp2, Inhbb, Nol3, Esr1*. Knock out, knock down, overexpression or a higher activity of these genes causes overall muscle hypertrophy as measured by an increased muscle weight or cross sectional area. The mean effect sizes range from 5 to 345% depending on the manipulated gene as well as the muscle size variable and muscle investigated. Bioinformatical analyses reveal that *Asb15, Klf10, Tpt1* are most highly expressed hypertrophy genes in human skeletal muscle when compared to other tissues. Many of the muscle hypertrophy-regulating genes are involved in transcription and ubiquitination. Especially genes belonging to three signaling pathways are able to induce hypertrophy: (a) Igf1-Akt-mTOR pathway, (b) myostatin-Smad signaling, and (c) the angiotensin-bradykinin signaling pathway. The expression of several muscle hypertrophy-inducing genes and the phosphorylation of their protein products changes after human resistance and high intensity exercise, in maximally stimulated mouse muscle or in overloaded mouse plantaris.

## Introduction

In humans, skeletal muscle mass, fiber numbers, fiber size, and strength vary greatly. In 18–29 year old women and men muscle mass is 34 ± 6% and 42 ± 4% of the whole body mass, respectively (Janssen et al., 2000). Females on average have a lower muscle mass than males (Janssen et al., 2000) which can partially be explained by low levels of the male sex hormone testosterone, which promotes muscle hypertrophy (Sinha-Hikim et al., 2002). Humans have over 600 muscles and within muscles the number of fibers and their cross sectional area differs greatly. For example, in the *vastus lateralis* Lexell et al. counted between 393,000 and 903,000 muscle fibers in nine males aged 15–22 years. The average area of type 1 and type 2 fibers per individual ranged from 2,146 to 6,279 μm^2^ and 2,142 to 5,535 μm^2^, respectively (Lexell et al., 1988). Similarly, in 1,121,088 males aged 16–25 years the mean elbow flexion strength was 387 ± 84 N, hand grip strength 616 ± 98 N and knee extension strength 569 ± 118 N, respectively. This means that ≈5% of individuals could extend their leg either with a maximal force of either <333 N or more than 805 N (Silventoinen et al., 2008), highlighting the large variation of strength. Muscle mass and function are additionally lost during normal aging (Mitchell et al., 2012) which has been termed sarcopenia (Rosenberg, 1997). In summary, muscle mass and function variables vary greatly in human populations and decline with normal aging.

What are the consequences of this large variation in muscle mass and function? Skeletal muscle is the largest organ in terms of percent of body mass. Muscle takes up circulating glucose, releases amino acids into the circulation during fasting, and low muscle strength is associated with an increased risk of falls (Wolfe, 2006). In a prospective study, researchers additionally found that strength was associated with a significantly higher all-cause and cancer mortality in both individuals below 60 years of age and above 60 years (Ruiz et al., 2008). Thus, muscle mass and function not only matter for athletic performance but also influence our health, how well we age and how long we live.

What factors influence muscle mass and strength and how much is this influenced by variations in the DNA sequence (i.e., genetics)? As almost all traits, muscle mass and function depend on both nature (i.e., genetics or DNA sequence variation) and nurture, which is environmental factors such as resistance training (American College of Sports Medicine, 2009) and nutrition. In twin and family/sib-pair studies researchers estimated the heritability of strength. The results varied greatly from 0.14 to 0.97 (Peeters et al., 2009) perhaps showing the limitations of studies aimed at estimating heritability. In the largest twin study, elbow, hand grip, and knee extension strength were estimated to be 56, 66, and 61% inherited, respectively (Silventoinen et al., 2008). In the extreme, patients with monogenetic muscle diseases can have very poor muscle function (Kaplan, 2011) whereas some elite athletes in strength and power sports have extreme muscle mass and function.

Transgenic mouse models have helped us to identify genes where variations in the DNA sequence cause disease or influence traits including muscle mass and function. Genetic modifications involve naturally occurring mutations or genetically engineered ones. In 2007 Mario R. Capecchi, Martin J. Evans and Oliver Smithies won the Nobel Prize in Physiology or Medicine “*for their discoveries of principles of introducing specific gene modifications in mice by the use of embryonic stem cells*” or short transgenic mice technology. In contrast to chemically induced random point mutation generation e.g., by ethylnitrosourea (ENU; Russell et al., 1979), transgenic methods are designed to make genes non-functional (i.e., knock out/down or loss-of-function) or to increase the function or expression of a gene (i.e., knock in or gain-of-function). The added DNA might be inserted randomly or to targeted sequences. The transgene construct could contain at minimum a defined promotor (widespread or skeletal muscle/tissue-specific), start and stop codons as well as ribosomal recognition sites, and typically also includes selection markers such as antibiotics resistance or reporter genes like β-galactosidase (lacZ). Gene targeting in mice makes use of homologous recombination which exchanges endogenous DNA with a modified DNA sequence. To further specify gene inactivation at a given time point or in defined tissues conditional mutagenesis is performed e.g., using the Cre/loxP recombination system (Nagy, 2000). Cre is a recombinase from the bacteriophage P1 that mediates excision of gene sequences between loxP sites introduced to the gene of interest by homologous recombination (Turan and Bode, 2011). There is a variety of tissue-specific and drug-inducible Cre lines available allowing temporal and spatial gene function analysis. The next generation of transgenesis methods makes use of engineered nucleases targeting directly the gene of interest thus reducing time and cost for mouse breeding. These include Zinc-finger nucleases (ZFNs; Maeder et al., 2008), Transcription Activator-Like Effector Nucleases (TALENs; Engler et al., 2008) and the CRISPR-Cas system (Cong et al., 2013; Mali et al., 2013). Especially CRISPR-Cas allows researchers to modify several genes which mimics multigenic human diseases and phenotypes. In addition, adeno-associated virus (AAV) vectors are used for *in vivo* gene transfer and are already applied for human gene therapy also in muscle (for review see Boisgerault and Mingozzi, 2015).

Transgenic methods in mice have led to the discovery of genes whose gain or loss-of-function results in muscle hypertrophy in mice. The most prominent example for this is the myostatin knock out mouse (gene symbol *Mstn* or *Gdf8*). A loss of *Mstn* in mice or humans both roughly doubles muscle mass (McPherron et al., 1997; Schuelke et al., 2004). Thus, genes whose experimentally induced mutation or change of expression/activity affects muscle mass or function in mice are “candidate genes” for muscle mass and function in humans.

To date, there is no systematic compilation of genes whose mutation causes muscle hypertrophy in mice. The aim of this systematic review was therefore to systematically search the literature for published studies where a gain or loss-of-function mutation of a gene induces skeletal muscle hypertrophy. Through this analysis we identify 47 genes whose mutation induces muscle hypertrophy in mice. Additionally, we performed bioinformatical analyses for these 47 genes to determine their expression pattern in human tissues and different muscle fibers as well as to find out whether these genes change their expression or become phosphorylated in response to high intensity and resistance (strength) exercise in human skeletal muscle, maximal mouse muscle contraction and synergist ablation-overloaded mouse plantaris muscle.

## Methods

### Systematic literature search

To identify publications that identify genes whose transgensis results in muscle hypertrophy, we carried out a systematic review according to the PRISMA guidelines (Moher et al., 2009) and searched the literature according to the PICO framework (Schardt et al., 2007). First, we searched PubMed (RRID:SCR_004846) using the following search terms: “mouse AND transgenesis AND (muscle mass OR muscle weight).” This search was repeated in PubReminer (http://hgserver2.amc.nl/cgi-bin/miner/miner2.cgi) to identify more relevant MeSH terms and keywords. From the PubReminer results we selected the search terms shown in Table S1. To narrow the number of studies, we added the search term “AND skeletal muscle” to exclude studies that reported hypertrophy of other tissues. Finally, we searched as follows: “((((((((mice) OR mouse) OR “mouse model”) OR mice transgenic)) AND ((((((gene transfer techniques) OR “overexpression”) OR “knockout”) OR mutagenesis) OR retroviridae) OR gene deletion)) AND (((((((((“muscle mass”) OR hypertrophy) OR “muscle weight”) OR “hypermuscular”) OR “muscle growth”) OR “muscle fiber size”) OR “cross sectional area”) OR hyperplasia) OR phenotype))) AND skeletal muscle.”

We included articles from peer-reviewed journals, written in English, which studied muscle size in gene-manipulated mouse models *in vivo*. Studies were eligible when no pathologies were reported for the duration of the study as we aimed to identify genes that can potentially be targeted for hypertrophy without causing disease. Also, to exclude confounding factors on muscle growth from, for example, regeneration or muscle loading, gene manipulation had to be the only intervention used in the study. Furthermore, a measure of muscle mass had to be reported (e.g., muscle weight, cross sectional area, muscle diameter, or fiber number). In the case a muscle mass-influencing gene was reported more than once in the literature, only the first mention was included.

We excluded studies where one or more of the following applied:

1. rat or *in vitro* study,
2. no transgenesis or double mutation or miRNA manipulation,
3. mice showed disease or pathologies or were older than 12 months,
4. no effect, no outcome measures, or muscle atrophy,
5. no use of a wildtype or other control group,
6. not first mention of effect of gene on muscle mass.

From every relevant study we extracted the following information: author, gene name, protein name, method of gene manipulation, output measure, muscle(s) studied, muscle size values for transgenic and control mice, difference between transgenic and control mice in percentage, age of mice, mouse strain, additional measurements, and remarks, and compiled this information in Supplementary Table S2. Often the output measure values did not appear in the text, but in a bar graph. In that case, we manually estimated the relative difference between transgenic and control mouse from the bar graph (indicated in Table 1 with “^*^”). Also, we adopted the official gene name from Uniprot (RRID:SCR_002380) or Entrez Gene (RRID:SCR_002473). Therefore, it is possible that the mentioned gene name varies from an alias used in the original publication. Finally, note that we write the gene name in lowercase when it is unclear if the source of the overexpressed gene was human or other.

**Table 1:** Genes whose transgenesis in mice increases skeletal muscle mass.

**Gene symbol**	**Protein name**	**Transgenesis**	**Outcome measure**	**% Increase**	**Reference**
*Ski*	Ski oncogene	Global overexpression	Muscle CSA	+ 248%	Sutrave et al., 1990
*FST*	Follistatin	Conditional overexpression	Muscle weight Mean fiber CSA Fiber number	+ 193–345% + 28% + 66%	Lee and McPherron, 2001
*Acvr2b*	Activin receptor type-2B	Conditional overexpression dominant-negative form (loss of function)	Muscle weight Mean fiber CSA Fiber number	+ 109–179% + 40% + 27%	Lee and McPherron, 2001
*Akt1*	Protein kinase B	Plasmid DNA overexpression Inducible conditional overexpression^a^	Mean fiber CSA Muscle weight^a^	+ 62% + 218%^a^ + 48–73%^a^	Bodine et al., 2001 Lai et al., 2004
*Mstn, Gdf8*	Myostatin (*Gdf8)*	Global knock out	Muscle weight Mean fiber CSA Fiber number	+ 102–162% + 14–49% + 86%	McPherron et al., 1997
*Klf10, Tieg1*	TGFB-inducible early growth response protein 1	Global knock out	Muscle weight Muscle CSA Mean fiber CSA Fiber number	+ 22–24% + 41–57% + 71–90% + 20–31%	Kammoun et al., 2016
*Rheb*	GTP-binding protein Rheb	Plasmid DNA overexpression	Mean fiber CSA	+ 64%	Goodman et al., 2010
*Igf1*	Insulin-like growth factor I	Global overexpression Conditional overexpression^2^	Mean fiber CSA Muscle weight^2^	+ 17–115% + 35–50%^*^^2^ + 25%^*^^2^	Coleman et al., 1995 Musarò et al., 2001
*Pappa*	Pappalysin-1	Conditional overexpression	Muscle weight Muscle CSA Mean fiber CSA	+ 55%^*^ + 35%^*^ + 30%^*^	Rehage et al., 2007
*Ppard*	Peroxisome proliferator-activated receptor delta	Conditional overexpression	Fiber number	+ 13–72%	Luquet et al., 2003
*Ikbkb*	Inhibitor of nuclear factor kappa-B kinase subunit beta	Conditional knock out	Fiber number	+ 50%	Bakkar et al., 2008
*FSTL3*	Follistatin-related gene protein	Conditional overexpression	Muscle weight	+ 3%−80%	Lee, 2007
*Atgr1a*	Type-1A angiotensin II receptor	Germline knock out	Muscle weight/body weight Mean fiber CSA Fiber number	+ 6% + 52% + 32%	Zempo et al., 2016
*Ucn3*	Urocortin-3	Global overexpression	Muscle weight Mean fiber CSA	+ 20–85%^*^ + 35%^*^	Jamieson et al., 2011
*Mcu*	Calcium uniporter protein, mitochondrial	AAV overexpression	Mean fiber CSA	+ 37–47%	Mammucari et al., 2015
*Junb*	Transcription factor jun-B	AAV overexpression	Mean fiber CSA	+ 41%	Raffaello et al., 2010
*Ncor1*	Nuclear receptor corepressor 1	Conditional knock out	Muscle weight/body weight Mean fiber diameter	+ 40%^*^ + 10–35%^*^	Yamamoto et al., 2011
*Gprasp1*	G-protein coupled receptor-associated sorting protein 1	Global overexpression	Muscle weight Fiber number	+ 35–45% + 36%	Monestier et al., 2012
*Grb10*	Growth factor receptor-bound protein 10	Global knock out	Muscle weight	+ 35% - 40%	Smith et al., 2007
*Mmp9*	Matrix metalloproteinase-9	Conditional overexpression	Muscle weight Mean fiber CSA	+ 15%^*^ + 20–50%^*^	Dahiya et al., 2011
*Dgkz*	Diacylglycerol kinase zeta	Plasmid DNA overexpression	Mean fiber CSA	+ 33%	You et al., 2014
*Ppargc1a4*	Peroxisome proliferator-activated receptor gamma coactivator 1-alpha isoform 4	Conditional overexpression Conditional overexpression AAV overexpression Plasmid DNA overexpression	Muscle weight/tibia length Mean fiber CSA	+ 10–20%^*^+ 10% + 60% + 28%	Ruas et al., 2012
*Smad4*	Mothers against decapentaplegic homolog 4	Conditional knock out	Muscle weight Fiber numbers	+ 30%^*^ + 45–55%^*^	Sartori et al., 2013
*Ltbp4*	Latent-transforming growth factor beta-binding protein 4	Conditional overexpression	Muscle weight/tibia length Muscle weight/body weight	+ 25%^*^ + 25–40%^*^	Lamar et al., 2016
*Bmpr1a, Alk3*	Bone morphogenetic protein receptor type-1a	AAV overexpression	Muscle weight	+ 20–35%	Sartori et al., 2013
*Crtc2*	CREB-regulated transcription coactivator 2	Inducible conditional overexpression	Muscle weight Mean fiber CSA	+ 10–15%^*^ + 25%^*^	Bruno et al., 2014
*XIAP*	E3 ubiquitin-protein ligase XIAP	Conditional overexpression	Mean fiber CSA	+ 25%^*^	Hu et al., 2010
*Dgat1*	Diacylglycerol O-acyltransferase 1	Global knock out	Muscle weight	+ 21–28%	Liu et al., 2011
*Thra*	p43	Global knock out	Muscle weight Mean fiber CSA	+ 12% + 23%	Pessemesse et al., 2012
*Adrb2*	Beta-2 adrenergic receptor	AAV overexpression	Muscle weight/tibia length Mean Feret's diameter	+ 22% + 10%^*^	Hagg et al., 2016
*Asb15*	Ankyrin repeat and SOCS box protein 15	Plasmid DNA overexpression	Mean fiber CSA	+ 15–27%	McDaneld et al., 2006
*Cast*	Calpastatin	Conditional overexpression	Muscle weight	+ 12–47%	Otani et al., 2004
*Eif2b5*	Translation initiation factor eIF-2B subunit epsilon	Plasmid DNA overexpression	Mean fiber CSA	+ 21%	Mayhew et al., 2011
*Bdkrb2, Bk2r*	B2 bradykinin receptor	Global knock out	Muscle weight/body weight	+ 10–30%^*^	de Picoli Souza et al., 2010
*Tpt1, Tctp*	Translationally-controlled tumor protein 1	Plasmid DNA overexpression	Mean fiber CSA	+ 17–22%	Goodman et al., 2017
*Nr3c1, Grl1*	Glucocorticoid receptor	Conditional knock out	Muscle weight/body weight	+ 10–25%^*^	Shimizu et al. (2015)
*Nr4a1, Nur77*	Nuclear receptor subfamily 4 group A member 1	Conditional overexpression	Mean fiber CSA	+ 5–25%^*^	Tontonoz et al., 2015
*Gnas^3^*	Guanine nucleotide-binding protein G(s) subunit alpha	AAV overexpression	Muscle weight/tibia length Mean Feret's diameter	+ 8–27%^3^ + 10%^*^	Hagg et al., 2016
*Pld1*	Phospholipase D1	AAV overexpression	Mean fiber CSA	+ 16%	Jaafar et al., 2013
*Crym*	Ketimine reductase mu-crystallin	Global knock out	Mean fiber CSA	+ 15%^*^	Seko et al., 2016
*Camkk1*	Calcium/calmodulin-dependent protein kinase kinase 1	Plasmid DNA overexpression	Muscle weight	+ 10%	Ferey et al., 2014
*Yap1*	Yes-associated protein 1	AAV overexpression	Muscle weight Mean fiber CSA	+ 13% + 10%	Watt et al., 2015
*Inhba*	Inhibin beta A chain; Activin beta-A chain	Germline knock out	Muscle weight	+ 5–7%	Lee et al., 2010
*Tp53inp2*	Tumor protein p53-inducible nuclear protein 2	Conditional knock out	Muscle weight	+ 5–10%^*^	Sala et al., 2014
*Inhbb*	Inhibin beta B chain; Activin beta-B chain	Germline knock out	Muscle weight	+ 6–7%	Lee et al., 2010
*Nol3*	Apoptosis repressor with CARD	Global knock out	Muscle CSA	−15%^§^+ 22%	Mitchell et al., 2015
*Esr1*	Estrogen receptor	Global knock out	Muscle weight Muscle weight/body weight	+ 15%^*^ – 10%^*^	Brown et al., 2009

*The 47 genes whose transgenesis increases muscle mass after gene manipulation are shown*.

* *Indicates that values are estimated from graph bars.^§^Gene manipulation increased (plantaris) and decreased (soleus) muscle CSA depending on muscle type. AAV Adeno-associated virus. CSA Cross-sectional area*.

a *Inducible conditional overexpression of Akt1 increases muscle weight by 48–73% and mean fiber CSA by 218% Lai et al. (2004). ^b^Musarò et al. (2001) validated the findings of Coleman et al. (1995) and observed a 25% increase in muscle weight and 35–55% increase in mean fiber CSA after conditionally overexpressing Igf1 in muscle. ^c^Two isoforms of Gnas were manipulated (large and extra large isoform) that are both included in the table. More extensive table found in Supplementary Table S2*.

### Bioinformatical analyses

To determine whether the muscle hypertrophy-inducing genes are expressed specifically in skeletal muscle or elsewhere, we retrieved expression figures from the Genotype-Tissue Expression (GTEx; RRID:SCR_001618; GTEx Consortium, 2015) database and pasted the figures into worksheet 1 in Supplementary Table S3.

To compare the expression of muscle hypertrophy-inducing genes in mouse type 1 and type 2b fibers, we downloaded the microarray dataset **GSE23244** (Chemello et al., 2011) from Gene Omnibus (RRID:SCR_007303) and retrieved the data with GEO2R. We then copied for all 47 muscle hypertrophy-inducing genes the adjusted *p-*values (adj.P.Val) and the log fold changes (logFC) into worksheet 2 in Supplementary Table S3. Positive logFC values indicate that a gene is more expressed in type 1 fibers. Negative logFC values indicate that the gene is more expressed in type 2b fibers.

To identify secreted hypertrophy-inducing genes we retrieved a list of genes/proteins that are predicted to be secreted from the Human Protein Atlas (https://www.proteinatlas.org/; Uhlén et al., 2015) and performed an overlap analysis using a web-based tool to identify genes in two lists (http://jura.wi.mit.edu/bioc/tools/compare.php).

To find out whether the 47 muscle hypertrophy-inducing genes interact through direct interaction of the proteins they encode or through functional interaction, we performed a STRING database analysis (Szklarczyk et al., 2015; https://string-db.org/; RRID:SCR_005223) which illustrates such interactions. The data are presented as an interaction figure and as a table in worksheet 3 in Supplementary Table S3.

To identify common functions and other links for the 47 muscle hypertrophy-inducing genes we performed bioinformatical enrichment analyses using DAVID (Huang da et al., 2009; https://david.ncifcrf.gov/summary.jsp; RRID:SCR_001881) and pasted the data into worksheets 4 and 5 in Supplementary Table S3. Subsequently we ordered the raw data so that functional categories and gene ontology, that were significantly enriched, are at the top of the list. For the functional enrichment analysis we used a background list of proteins that are expressed in skeletal muscle (Deshmukh et al., 2015; worksheet 6 in Supplementary Table S3).

To see if the identified genes are linked to human phenotypes we used the GWAS catalog (MacArthur et al., 2017; https://www.ebi.ac.uk/gwas/; RRID:SCR_012745), a website that allows to query Genome-wide association studies.

To study whether hypertrophy-inducing genes change their expression in skeletal muscle after resistance (strength) or endurance exercise, we downloaded the transcriptome microarray dataset **GSE59088** (Vissing and Schjerling, 2014) from Gene Omnibus (https://www.ncbi.nlm.nih.gov/geo/; RRID:SCR_007303) and plotted the gene expression data 2.5 h and 5 h after human resistance exercise in relation to pre exercise.

To see how the expression of muscle hypertrophy-inducing genes changes during overload-induced hypertrophy in synergist-ablated mouse plantaris muscle, we retrieved the microarray dataset **GSE47098** data from Chaillou et al. (2013). We copied and pasted for all 47 hypertrophy-inducing genes the data into worksheet 7 of Supplementary Table S3 and calculate their expression relative to the unstimulated plantaris (day 0).

We also investigated whether hypertrophy-inducing genes change their phosphorylation after exercise. For this, we downloaded supplementary data from two phosphoproteome studies. The first investigated protein phosphorylation changes after a single bout of high intensity training in human muscle (Hoffman et al., 2015, Supplementary Table S1 of that paper). The second studied protein phosphorylation in mouse skeletal muscle after electrically evoked maximal-intensity contractions (Potts et al., 2017; supporting information file tjp12447-sup-0001-Table S1.xlsx). We downloaded the supplementary files mentioned and copied and pasted the relevant data into worksheet 8 of Supplementary Table S3.

## Results

We searched PubMed using our systematic search strategy and identified 1,982 papers with publication dates until June 2017. Based on the title or abstract we excluded 1,861 studies. After this 132 articles remained that were assessed full-text for eligibility. Twenty-seven more articles were identified by reviewing the reference lists of the full-text articles or other sources. Finally, we read 159 full-text and analyzed 45 articles quantitatively. The PRISMA flowchart describing our search and selection is in the Supplementary Material (Figure S1).

### Gene manipulations that result in skeletal muscle hypertrophy

**Figure 1:**
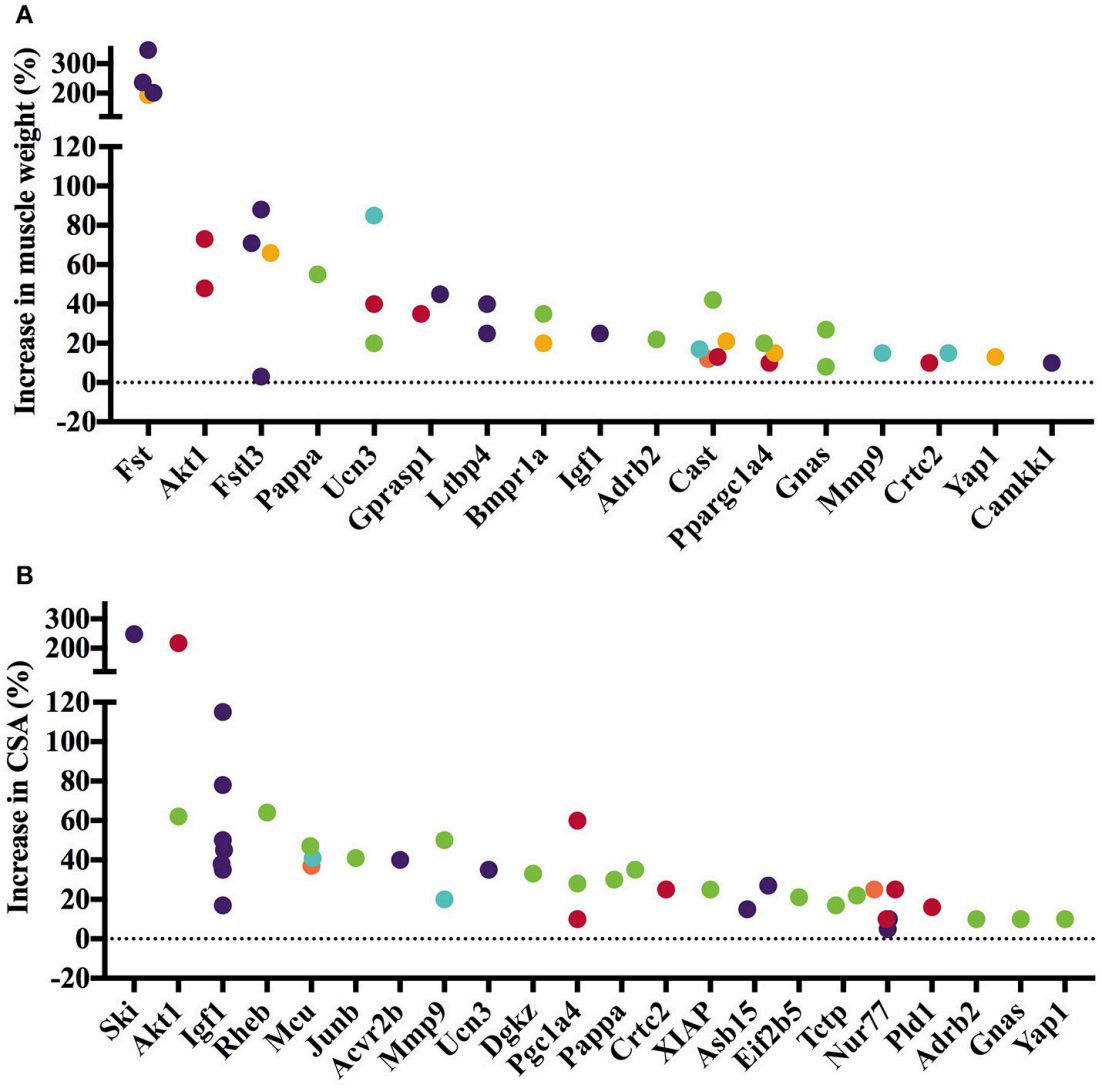
Gene knock-in or overexpression increases muscle weight and cross-sectional area (CSA). Increase in muscle weight **(A)** and fiber or muscle CSA **(B)** differs across genes and between muscles. Individual muscles are color-coded as follows: • Gastrocnemius, • Soleus, • Tibialis anterior, • Quadriceps, • Extensor digitorum longus, • Other. Data is based on values collected in Supplementary Table S2.

**Figure 2:**
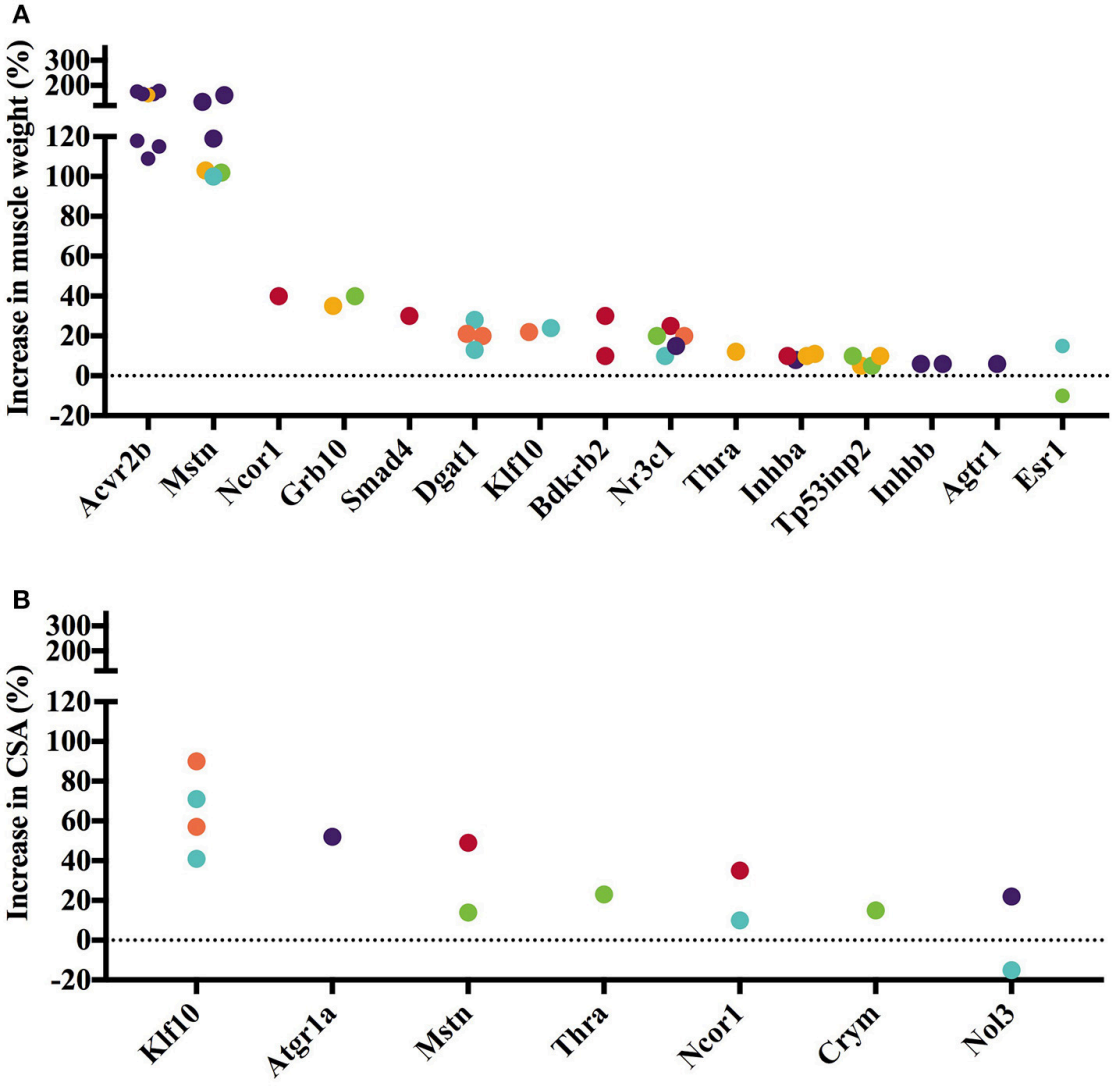
Gene knock out or loss-of-function increases muscle weight and cross-sectional area (CSA). Increase in muscle weight **(A)** and fiber or muscle CSA **(B)** after gene knock out differs across genes and between muscles. Individual muscles are color-coded as follows: • Gastrocnemius, • Soleus, • Tibialis anterior, • Quadriceps, • Extensor digitorum longus, • Other. Data is based on values collected in Supplementary Table S2.

The 45 analyzed articles report 47 genes whose gain or loss-of-function through transgenesis increased skeletal muscle mass significantly between 5 and 345% (Table 1). To illustrate the muscle hypertrophy-inducing effect of transgenesis in different muscles, we plotted increases in muscle weight per muscle (Figures 1A, 2A), as well as increases in muscle or myofiber cross sectional area (Figures 1B, 2B). Figure 1 shows genes whose gain-of-function increases muscle mass and Figure 2 reports genes whose loss-of-function increases muscle mass. Overexpression of *Fst* and *Ski* most increased muscle weight (Figure 1A) and fiber cross sectional area (CSA; Figure 1B), respectively. In contrast, among the knock out genes the loss-of function of *Acvr2b* and *Mstn* increased muscle mass most (Figure 2A). The extent of muscle hypertrophy can vary greatly after transgenesis of the same gene. For example, *Ucn3* overexpression increases the muscle weight of the soleus by 85% but in the tibialis anterior (TA) only by 20%. Intriguingly, knock out of *Esr1* or *Nol3* increased muscle mass of the soleus but caused atrophy of the TA or plantaris, respectively (Figures 2A,**B**).

### In what tissues and in what muscle fibers are muscle hypertrophy-associated genes expressed?

To find out whether the muscle hypertrophy-associated genes are mainly expressed selectively in skeletal muscle, we retrieved tissue-specific gene expression data from the GTEx Portal database (GTEx Consortium, 2015; worksheet 1 in Supplementary Table S3). This analysis revealed that *Asb15, Klf10*, and *Tpt1* were the only genes that were most expressed in skeletal muscle when compared to other human tissues. The genes *Cast, Mcu, Mstn, Nol3*, and *Ppargc1a* were highly expressed in skeletal muscle (top 10 of all tissues) whereas the remainder of the genes were generally more expressed in tissues other than skeletal muscle. Together this suggest that only a minority of muscle hypertrophy genes are genes that are highly or specifically expressed in skeletal muscle.

Skeletal muscle is a heterogeneous tissue comprising of slow type 1, intermediate type 2a, and fast 2x and 2b (only in rodents) muscle fibers. To test for muscle fiber-specific gene expression, we retrieved the transcriptome microarray dataset **GSE23244** from Gene Omnibus that reported gene expression levels for mouse type 1 and type 2b muscle fibers (Chemello et al., 2011). We could not find expression data for 13 genes. Of the remaining genes, *Mstn, Gnas* and *Nol3* were differently expressed in-between type 1 and type 2b fibers with Mstn being 20-fold more expressed in type 2b fibers whereas *Gnas* and *Nol3* are significantly but moderately more expressed in type 1 fibers.

### How many hypertrophy genes are predicted to be secreted?

To identify genes that encode proteins that are predicted to be secreted we downloaded a list of secreted proteins from the Human Protein Atlas (Uhlén et al., 2015) and overlapped them with the list of hypertrophy-inducing genes. This revealed that 11 out of the 47 genes are predicted to be secreted (*Fst, Fstl3, Gnas, Igf1, Inhba, Inhbb, Ltbp4, Mmp9, Mstn, Pappa, Ucn3*).

### Do muscle hypertrophy genes interact?

We performed a STRING analysis to detect functional association between the 47 hypertrophy-associated genes. This is defined as “*a specific and productive functional relationship between two proteins, likely contributing to a common biological purpose*” (Szklarczyk et al., 2015). This analysis revealed multiple functional associations that are illustrated in worksheet 3 of Supplementary Table 3. One important functional association cluster is linked to myostatin-Smad signaling and includes the genes *Mstn, Fst, Fstl3, Inhba, Inhbb, Acvr2b, Bmpr1a, Smad4*, and *Ski*. Another cluster comprises genes linked to the Igf1-Akt-mTOR signaling network and includes *Igf1, Akt1*, and *Rheb* as central members of this network plus other genes that encode proteins that functionally associate. Finally, *Agtr1a, Bdkrb2, Adrb2*, and *Gnas* are linked to angiogensin-bradykinin and G-protein coupled receptor signaling. Together this suggests that hypertrophy-regulating genes belong to several signaling systems that are capable of inducing skeletal muscle hypertrophy.

### Do the muscle hypertrophy genes have similar functions?

To learn more about the biological functions of the 47 muscle mass-increasing genes in mice, we performed functional enrichment analyses using DAVID (Huang da et al., 2009; RRID:SCR_001881). This analysis revealed that 28% of the muscle hypertrophy-inducing genes are associated with ubiquitination and 21% regulates transcription (worksheet 4 and 5 in Supplementary Table S3).

### Are muscle hypertrophy genes linked to human phenotypes in GWAS studies?

To see if the identified genes are linked to human phenotypes we used the Genome Wide Association Studies (GWAS) catalog (MacArthur et al., 2017; RRID:SCR_012745). No muscle phenotypes in humans are linked with the genes we found. Still, some muscle and growth related associations are found. *PPARD, PAPPA*, and *ESR1* associate with height (Allen et al., 2010; Wood et al., 2014) and *PLD1* is linked to the body mass index (BMI Ng et al., 2012). Also, *Tpt1*, which is highly expressed in skeletal muscle, is associated with type 2 diabetes (Anderson et al., 2015).

### Do muscle hypertrophy genes change their expression in response to strength (resistance) exercise?

**Figure 3:**
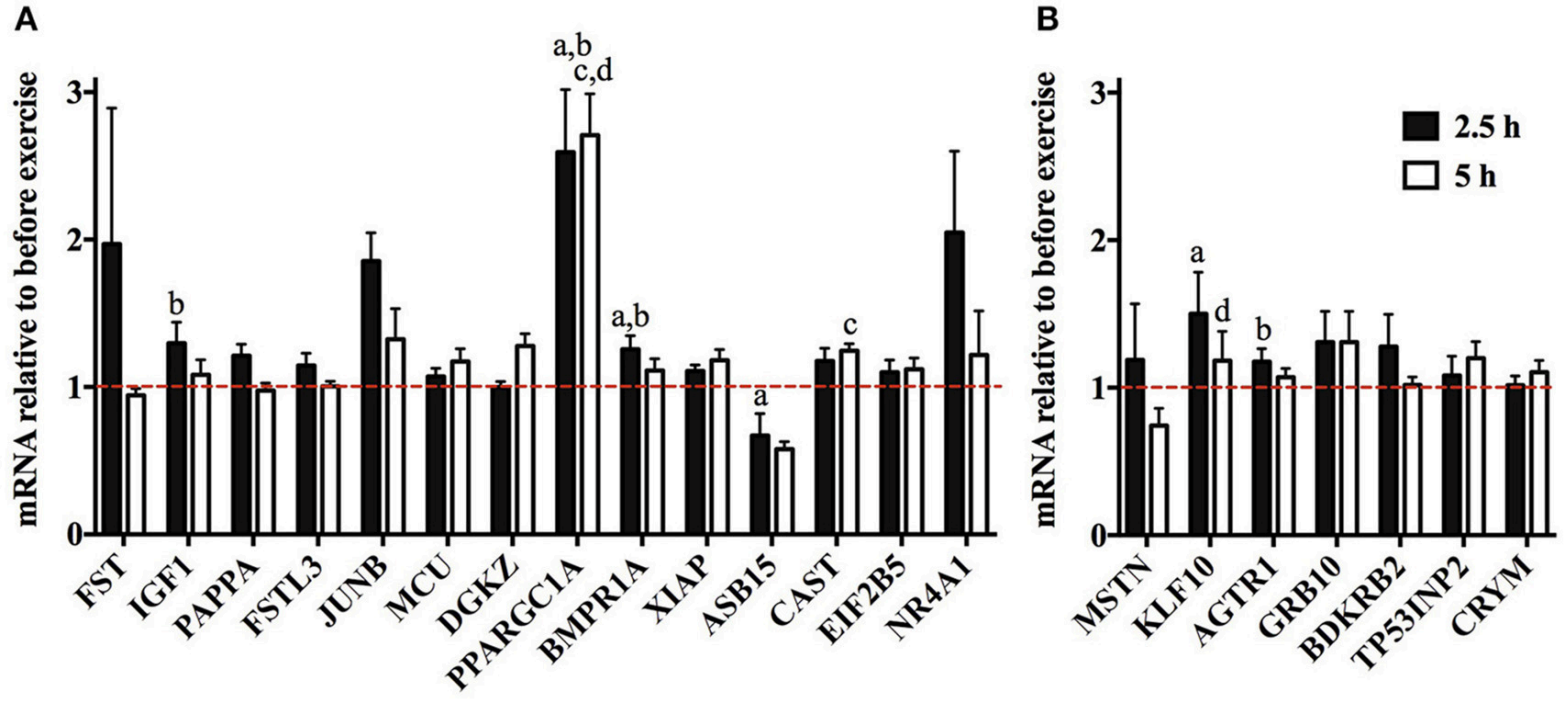
Expression of muscle mass regulating genes after resistance exercise. Transcriptome data GSE23244 from Vissing and Schjerling (2014) was accessed to discover which muscle mass regulating genes are induced or repressed 2.5 h or 5 h relative to resting value after a bout of resistance exercise. **(A)** Expression of genes whose overexpression or activation increases muscle mass in mice. **(B)** Expression of genes whose knock out increases muscle mass. Genes whose mean expression increased or decreased by more than 10% are shown in this figure. ^a^Different at 2.5 h compared to pre-training, ^b^Different compared to control group at 2.5 h, ^c^Different at 5 h compared to pre-training, ^d^Different compared to control group at 5 h (*n* = 6). Note that the data for pan *PPARGC1A* are shown. However, muscle hypertrophy is only stimulated by the PGC-1α4 protein isoform (Ruas et al., 2012).

To find out whether the muscle hypertrophy-causing genes are induced or repressed by resistance exercise in humans, we accessed transcriptome data from Vissing and Schjerling (2014). This revealed that the expression of some of these genes (*IGF1, PPARGC1A, BMPR1A, ASB15, CAST, KLF10*, and *AGTR1*) changes significantly by more than 10% after resistance exercise in human muscle (Figure 3).

### Does the expression of hypertrophy genes change during overload-induced hypertrophy in a way that is consistent with their function?

**Figure 4:**
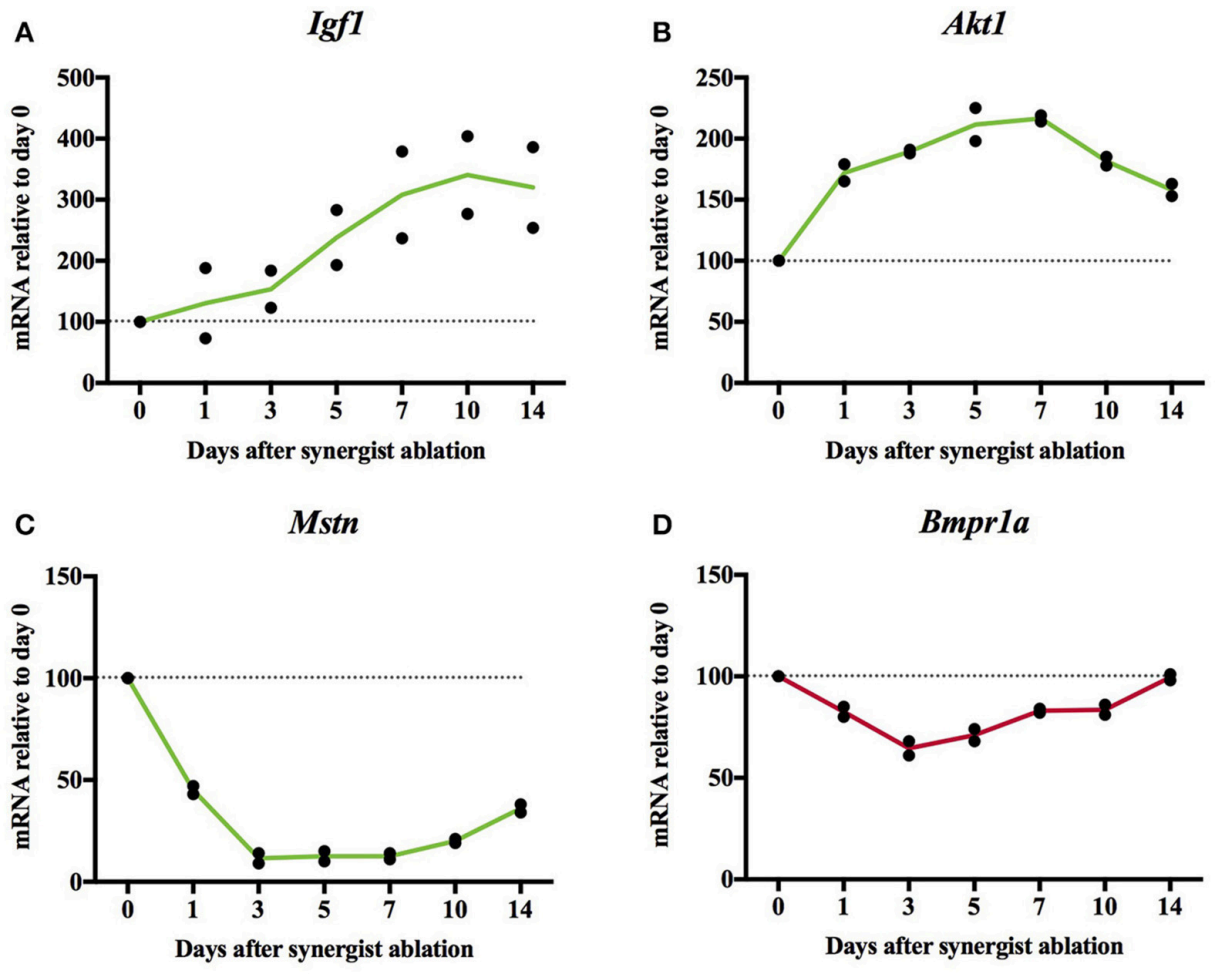
Relative mRNA expression of *Igf1* **(A)**, *Akt1* **(B)**, *Mstn* **(C)**, and *Bmpr1a* **(D)** in synergist ablation-overloaded mouse plantaris muscle. *Igf1, Akt1, Mstn* change their expression which is consistent with the hypertrophy of the plantaris. In contrast, *Bmpr1a* did not change its expression as expected as the overexpression of *Bmpr1a* causes hypertrophy (Sartori et al., 2013) whilst its expression decreased in the hypertrophying plantaris (see Supplementary Table S3, worksheet 7 for further figures). Original data derived from microarray dataset **GSE47098** data from Chaillou et al. (2013).

To find out how the expression of muscle hypertrophy-increasing genes changes in a mouse plantaris that is hypertrophying because of synergist ablation, we re-analyzed transcriptome data from Chaillou et al. (2013). We expected increased expression of genes whose gain-of-function causes muscle hypertrophy and decreased expression of genes whose loss-of-function causes hypertrophy. Generally, genes whose change-of-function has a large effect on muscle size changed their expression as expected (e.g., *Acvr2b, Akt1, Fst, Fstl3, Igf1*, and *Mstn*; Figure 4; Supplementary Table S3, worksheet 7). Of these genes, *Mstn* expression was one of the genes with the largest drop of expression in the hypertrophying plantaris muscle genome wide. Overall, only roughly half of the hypertrophy-inducing genes changed their expression as expected whereas the other half did change their expression in an unexpected way or their expression remained stable.

### Do hypertrophy-inducing proteins change their phosphorylation after human high-intensity exercise and maximal mouse muscle contraction?

Protein phosphorylation is a common mechanism of signal transduction that is essential for many skeletal muscle adaptations to exercise. To test whether hypertrophy-inducing proteins are phosphorylated in muscle and whether their phosphorylation changes in response to human high intensity exercise or maximal mouse muscle contraction, we re-analyzed the phosphoproteomic datasets of Potts et al. (2017) and Hoffman et al. (2015) (Supplementary Table S3, worksheet 8). In their non-biased analyses the authors detected phosphoproteins for the hypertrophy-inducing genes *ADRB2, AKT1, CAMKK1, Cast/CAST, CRTC2, Dgkz/DGKZ, Eif2b5, GNAS, GRB10, Ltbp4, Nr3c1, Pld1, and Yap1/YAP1* (lower cases denote the mouse gene symbols from (Potts et al., 2017) and the upper case gene symbols the human gene symbols from Hoffman et al. (2015). However, only the proteins encoded by *Cast/CAST, CAMKK1, CAST*, and *DGKZ* significantly changed their phosphorylation in response to mouse muscle stimulation or human high intensity exercise.

## Discussion

Through a systematic literature search we identified 47 genes whose genetic manipulation results in significant skeletal muscle hypertrophy in mice when compared to wildtype controls confirming that muscle mass and muscle hypertrophy is a polygenic trait. Of these 47 genes, 18 had to be knocked out (loss-of-function) and 29 were knocked in or overexpressed (gain-of-function) to induce muscle hypertrophy. This shows that muscle mass is regulated by both muscle growth factors and muscle mass inhibitors, which have been termed “chalones” (Lee, 2004). The 47 hypertrophy-inducing genes are candidate genes that encode proteins that are potentially involved in developmental growth, adult muscle mass, hypertrophy in response to resistance (strength) training and feeding, and their dysregulation might contribute to the loss of muscle mass and function during normal aging, termed sarcopenia (Rosenberg, 1997) as well as several other forms of atrophy.

The first example where one of these genes has served as a candidate gene for a human genetic discovery is the *Mstn* (McPherron et al., 1997) which served as a candidate gene for discoverig a homozygous, intronic *MSTN* mutation in a hypermuscular child (Schuelke et al., 2004). However, Mstn mutants are also a good example to highlight that increased muscle size does not always translate into proportionally improved muscle function. For example, *Mstn* loss-of-function mice have more muscle mass but are not stronger than wildtype mice. The consequence is a reduced force-to-muscle mass ratio or muscle quality (Amthor et al., 2007). However, the relation between muscle mass and function appears to be species dependent as *Mstn* variants in whippet dogs are associated with increased running performance when compared to wildtype dogs (Mosher et al., 2007). Also the anecdotal evidence from the human MSTN knockout suggests that *MSTN* loss-of-function carriers had not only increased muscle mass but also more strength (Schuelke et al., 2004).

To further study the 47 muscle hypertrophy-inducing genes, we performed several bioinformatical analyses to learn more about the tissue-specific expression, functional association, and phenotype association as well as their response to resistance (strength) exercise, and synergist ablation-induced hypertrophy. Together this dataset gives an update on the genetics of skeletal muscle hypertrophy that adds to narrative reviews on the regulation of skeletal muscle mass (Schiaffino et al., 2013; Egerman and Glass, 2014; Marcotte et al., 2015). Whilst this is a systematic review, it is not unbiased as the authors of the original papers subjectively chose genes for mouse transgenesis. Here, the International Mouse Phenotyping Consortium (IMPC) might help to extend the list of hypertrophy-inducing genes in a less biased fashion. The aim of the IMPC is to generate knock out mice for 20.000 known or predicted mouse genes (http://www.mousephenotype.org/). These knock out mice are subjected to an extensive pipeline of phenotyping tests in order to discover the biological functions of each gene (Brown and Moore, 2012; Dickinson et al., 2016). In the IMPC phenotype pipeline skeletal muscle hypertrophy, muscle mass and fiber size are not directly measured but investigations of embryos, body weight, body composition as well as grip strength are phenotyping measures that can suggest skeletal muscle hypertrophy which can then be followed up with a specific skeletal muscle analysis.

One key question is whether the muscle hypertrophy associated genes belong to one major muscle hypertrophy pathway, to several, or whether most genes are functionally unrelated. To answer this question, we bioinformatically assessed human tissue-specific (GTEx Portal) and mouse muscle fiber-specific gene expression (Chemello et al., 2011), functional associations (STRING analysis) as well as a functional enrichment analysis using DAVID to identify commonalities among hypertrophy-inducing genes (Huang da et al., 2009). Only three genes (*Asb15, Klf10, Tpt1*) are most expressed in skeletal muscle and of all 47 genes only *Asb15* seems to be selectively expressed in skeletal and cardiac muscle. *Mstn* is generally expressed at low levels and selectively in fast type 2 muscle fibers (Chemello et al., 2011). Most other genes are most expressed in tissues other than muscle. Collectively this suggests that muscle hypertrophy-inducing genes are rarely specifically expressed in skeletal muscle but are more often expressed in multiple tissues or mainly in other tissues. This is important information for identifying muscle atrophy drug targets as the targeting of skeletal muscle-specific genes and proteins makes side effects less likely. Therapeutically relevant is also the finding that 11 out of the 47 genes are predicted to be secreted (*Fst, Fstl3, Gnas, Igf1, Inhba, Inhbb, ltbp4, Mmp9, Mstn, Papa, Ucn3*) as secreted proteins can be targeted better than intracellular proteins.

Analyzing functional associations with the functional association search programme STRING points to three pathways whose genes can induce muscle hypertrophy. They are the (a) Igf1-Akt-mTOR (Marcotte et al., 2015), (b) myostatin-Smad (Sartori and Sandri, 2015), and (c) angiotensin-bradykinin signaling pathways. Whilst it is extensively shown that Igf1-Akt-mTOR and myostatin-Smad signaling regulate muscle mass, there is less information on a link between angiotensin-bradykinin signaling and muscle mass. In humans, the angiotensin-converting enzyme ACE D deletion allele was associated with greater strength gain after resistance training but the study only involved 33 subjects (Folland et al., 2000). Also, an ACE inhibitor attenuated load-induced muscle hypertrophy in rats (Gordon et al., 2001) suggesting that the angiotensin-bradykinin system is involved in regulating muscle size. Still, Igf1-Akt-mTOR, myostatin-Smad and angiotension-bradykinin signaling are not the only pathways contributing to muscle growth. A growth stimulus from resistance, or high intensity exercise, increases transcription of many genes that do not have a direct link with these pathways (Vissing and Schjerling, 2014; Hoffman et al., 2015).

In addition to the 47 genes we identified, *IGSF1* might also be a candidate gene for rare and common DNA variants influencing muscle mass. Recently, a gene wide association study has linked DNA variation of the *IGSF1* gene to body size in dog breeds (Plassais et al., 2017). This is of interest because a single nucleotide variation in the *IGSF9B* locus was associated with human hand grip strength (Willems et al., 2017).

Resistance (strength) exercise is a key intervention to promote skeletal muscle hypertrophy (Schoenfeld, 2010). To find out whether the 47 muscle hypertrophy-inducing genes are potential regulators of the muscle hypertrophy response to resistance exercise, we re-analyzed a dataset of gene expression data obtained pre, 2.5 and 5 h after a bout of resistance exercise (Vissing and Schjerling, 2014). This revealed that *IGF1, PPARGC1A, BMPR1A, ASB15, CAST, KLF10*, and *AGTR1* are significantly altered by resistance exercise (Figure 3; note that muscle hypertrophy is only stimulated by the PGC-1α4 isoform Ruas et al., 2012). Interestingly, while *KLF10* and *AGTR1* expression increases after a single bout of resistance exercise, we identified that knock out of these genes in mice increases muscle mass (Kammoun et al., 2016; Zempo et al., 2016). Similarly, *ASB15* is repressed by resistance exercise and in the synergist ablation-overloaded plantaris whilst its overexpression results in muscle hypertrophy in mice (McDaneld et al., 2006). Thus, the direction of the change of the expression of hypertrophy-inducing genes at 2.5 and 5 h after resistance exercise is not always consistent with their function.

A chronic overload-induced muscle hypertrophy model is synergist ablation. Here, we re-analyzed the data of the synergist ablation-overloaded plantaris from Chaillou et al. (2013) which measured gene expression at days 1, 3, 5, 7, 10, and 14 which gives a detailed time course of gene expression during a hypertrophy stimulus. We found that genes such as *Akt1, Igf1, and Mstn* whose gain-or-loss of function has a large effect on muscle size changed their expression as predicted. This suggests that many hypertrophy-inducing genes contribute to synergist ablation-induced hypertrophy through a regulation of their gene expression. However, other genes such as *Bmpr1a* (Figure 4), change their expression not as expected (e.g., increases of the expression of genes whose loss-of-function causes muscle hypertrophy) or remain relatively stable. Some hypertrophy-inducing proteins are also phosphorylated in skeletal muscle and the proteins encoded by *Cast/CAST, CAMKK1, CAST*, and *DGKZ* significantly change their phosphorylation in response to high intensity exercise or maximal muscle contraction.

Thalacker-Mercer et al. (2013) compared gene expression with the response to resistance exercise and found that the hypertrophy-inducing genes *DGKZ, MSTN, IGF1, ESR1, ACVR2Bb, SKI*, and *AKT1* are differentially expressed in extreme responders vs. non-responders. Genetic variability between individuals likely determines the muscle adaptation to exercise. For instance, it has been shown that gains in lean mass, muscle fiber/muscle hypertrophy, and power in humans are associated with DNA sequence variants of the ACE gene (Montgomery et al., 1999; Valdivieso et al., 2017).

Limitations of this systematic review are the strict inclusion and exclusion criteria determined before collecting the relevant literature. As a result of our search strategy we might have missed relevant studies. To identify relevant papers for our review we initially only screened abstracts and titles. Studies that observed a muscle phenotype, but failed to report this in the title or abstract are possibly overlooked. We decided using only PubMed to identify papers for our review, however, additional databases could have given extra results. We excluded double knock out studies which sometimes can provide important insight. For example, the combined knock out of *Mstn* and the expression of a *Fst* transgene roughly quadrupled muscle weight suggesting that the hypertrophy-inducing genes can have additive effects (Lee, 2007). Also we did not include chemically induced or naturally occurring mutants nor did we include studies where muscle hypertrophy was accompanied by a pathological phenotype. Note, that although we excluded genes whose manipulation was confounded by a pathology, we acknowledge that in this case the manipulated gene can still play a role in muscle hypertrophy. A follow-up literature evaluation for the identified hypertrophy genes was not performed. We only assessed the original papers that report an effect on muscle mass after gene gain or loss-of-function. For example, we did not assess whether studies other than the original one, report pathologies such as increased fibrosis or decreased life span. Most muscle phenotypes are determined at 2–3 months of age (Supplementary Table S2) and decreased life span for example would not become apparent. Also, we do not know whether the increases in muscle mass reported in the studies are maintained through life.

## Summary and conclusion

In summary, we found 47 genes whose transgenesis in mice results in muscle hypertrophy. These genes are candidate genes for rare and common DNA variants influencing human muscle mass. They also encode proteins that are potential targets for muscle addressing drug discovery, especially when secreted or expressed in a muscle-specific fashion. Most hypertrophy-inducing genes do not appear to change their expression or phosphorylation in a hypertrophy-promoting direction in the minutes and hours after human resistance and high intensity exercise. This is different, however, during day 1–14 of plantaris overload through synergist ablation. Here, especially hypertrophy-inducing genes with a large effect size such as *Akt1, Igf1, and Mstn* change their expression in a direction that is consistent with their involvement in hypertrophy regulation (Chaillou et al., 2013).

## Author contributions

HW and LB conceptualized review. SV and FN conducted systematic literature search. SV performed literature analysis. MS screened bioinformatic databases. SV and HW drafted manuscript. SV, HW, LB, and MHdA edited manuscript.

### Conflict of interest statement

The authors declare that the research was conducted in the absence of any commercial or financial relationships that could be construed as a potential conflict of interest.
