# Supplementary material for: Genes Whose Gain or Loss-Of-Function Increases Skeletal Muscle Mass in Mice: A Systematic Literature Review
Source: Front Physiol. 2018 May 22;9:553. doi: 10.3389/fphys.2018.00553 (PMC5992403; doi:10.3389/fphys.2018.00553)
Supplement: Supplementary file 1 [file Data_Sheet_1.docx]

Supplementary Material

Genes whose gain or loss-of-function increases skeletal muscle mass in mice: a systematic literature review

Sander AJ Verbrugge, Martin Schönfelder, Lore Becker, Fakhreddin Yaghood Nezhad, Martin Hrabě de Angelis, Henning Wackerhage^*^

*** Correspondence:**Prof. Dr. Henning Wackerhage
henning.wackerhage@tum.de

| **Table S1. Full list of PICO (Population, intervention, comparison, outcome) search terms** | | | |
| --- | --- | --- | --- |
| **Population** | **Intervention** | **Comparison** | **Outcome** |
| Mice (MeSH) | Gene transfer techniques (MeSH) | Wild type or Control | Muscle mass (KW) |
| Mice, transgenic (MeSH) | Overexpression (KW) |  | Hypertrophy (MeSH) |
| Mouse model (KW) | Knockout (KW) |  | Muscle weight (KW) |
| Mouse (KW) | Mutagenesis (MeSH) |  | Hypermuscular (KW) |
| Murine | Retroviridae (MeSH) |  | Muscle growth (KW) |
|  | Gene deletion (MeSH) |  | Muscle fiber size (KW) |
|  |  |  | Cross sectional area (KW) |
|  |  |  | Hyperplasia (MeSH) |
|  |  |  | Phenotype (MeSH) |
|  | | | |

We used the PICO framework for our systematic review to identify relevant studies. Table 1 shows MeSH terms and key words (KW) we used for the systematic literature search in Pubmed.

## Identification

Records identified through PubMed search (n= 1982)

Full-text articles excluded, with reasons (n = 101)

- No mouse model

- Early death, disease or abnormalities

- No or double gene manipulation

- No control

- No outcome measures, or muscle atrophy

- Not first mention

Records excluded
(n =1851)

Records after duplicates removed
(n = 1982)

## Screening

Full-text articles identified from reviewing references or other sources
(n = 27)

Records screened
(n = 1982)

Full-text articles assessed for eligibility
(n = 159)

## Eligibility

Studies included in quantitative synthesis
(n = 45)

## Included

**Supplementary Figure S1. PRISMA Flow diagram**
